# Classification of Solid Oxide Fuel Cells

**DOI:** 10.3390/nano12071059

**Published:** 2022-03-24

**Authors:** Kairat A. Kuterbekov, Alexey V. Nikonov, Kenzhebatyr Zh. Bekmyrza, Nikita B. Pavzderin, Asset M. Kabyshev, Marzhan M. Kubenova, Gaukhar D. Kabdrakhimova, Nursultan Aidarbekov

**Affiliations:** 1 Faculty of Physics and Technical Sciences, L.N. Gumilyov Eurasian National University, Nur-Sultan 010008, Kazakhstan; kkuterbekov@gmail.com (K.A.K.); kubenova.m@yandex.kz (M.M.K.); gkabdrakhimova@yandex.kz (G.D.K.); nursultan02_22.10.92@mail.ru (N.A.); 2 Republican Public Association “Physical and Technical Society”, Nur-Sultan 010008, Kazakhstan; 3 Institute of Electrophysics, Ural Branch, Russian Academy of Sciences, 620016 Yekaterinburg, Russia; nipavzderin@iep.uran.ru

**Keywords:** solid oxide fuel cell, electrolyte-free fuel cells, proton-conducting electrolyte SOFC, single-chamber SOFC, direct-flame SOFC, microtubular SOFC

## Abstract

Solid oxide fuel cells (SOFC) are promising, environmentally friendly energy sources. Many works are devoted to the study of materials, individual aspects of SOFC operation, and the development of devices based on them. However, there is no work covering the entire spectrum of SOFC concepts and designs. In the present review, an attempt is made to collect and structure all types of SOFC that exist today. Structural features of each type of SOFC have been described, and their advantages and disadvantages have been identified. A comparison of the designs showed that among the well-studied dual-chamber SOFC with oxygen-ion conducting electrolyte, the anode-supported design is the most suitable for operation at temperatures below 800 °C. Other SOFC types that are promising for low-temperature operation are SOFC with proton-conducting electrolyte and electrolyte-free fuel cells. However, these recently developed technologies are still far from commercialization and require further research and development.

## 1. Introduction

The growing global energy demand coupled with the need to reduce emissions of environmentally harmful greenhouse gases have resulted in the search for new clean alternative energy sources. In this regard, fuel cells are attracting great attention. These are efficient and silent electrochemical devices that directly convert the chemical energy of a fuel into electrical energy without the limitations of the Carnot cycle. There are several types of fuel cells: alkaline fuel cells (AFC), phosphoric acid fuel cells (PAFC), molten carbonate fuel cells (MCFC), proton exchange membrane fuel cells (PEMFC), and solid oxide fuel cells (SOFC) [1]. The high operating temperature of SOFC (400–1000 °C) gives them certain advantages over other types of fuel cells. SOFC can use a wide range of hydrocarbons as fuel. Catalysts based on noble metals (for example, Pt) are not required for SOFC operation. Waste heat can be reused by cogeneration, which increases the overall efficiency of the system based on SOFC up to 90% [2]. In addition, all SOFC components are made of hard materials; therefore, they are not limited to plane geometry and can be shaped to any form.

Intensive research of SOFC has been going on for three decades. During this time, many electrolyte and electrode materials have been studied, and a large number of SOFC configurations have been proposed and implemented. The first works [3,4] in which the SOFC classification was considered divided the fuel cells according to their geometry. Subsequently, the initial classification was complicated [5,6], and the division of the cells into groups began to be carried out according to several criteria: temperature, form, supporting component, etc. However, a number of concepts such as single-chamber SOFC [7] and electrolyte-free fuel cell [8] are not considered in [5,6]. Although the standard criteria are applicable for these concepts, new division parameters must be introduced to correctly and unambiguously describe all SOFC designs. The expansion of the existing classification will make it possible to order the data on the features of each SOFC type and facilitate orientation in their diversity. In addition, the systematization of SOFC designs will help to identify unused versions and possibly indicate ways to solve technological problems by combining or adopting approaches used in different SOFC configurations.

In the presented work, we tried to collect all currently existing types of SOFC and highlight their advantages and disadvantages.

## 2. Classification of SOFC

The SOFC classification will be carried out according to several criteria: presence/absence of electrolyte, gas spaces separation, operating temperature, support types, and cell design. Individual fuel cells but not stacks will be used as the subject of classification, although it should be recognized that in many cases the advantages and disadvantages of a particular design are manifested precisely when cells are assembled into a stack.

### 2.1. Classification according to the Presence/Absence of Electrolyte

It is considered that the conventional SOFC structure is a three-layer one consisting of porous electrode layers (anode and cathode) separated by a dense electrolyte layer. However, in the last decade, electrolyte-free fuel cells (EFFC) have been developed. Sometimes, they are also called electrolyte-layer-free fuel cells, single-component fuel cells (SCFC), or non-electrolyte separator fuel cells (NEFC).

Three-layer solid oxide fuel cells (TL-SOFC) are divided into two large classes according to the type of charge carrier in the electrolyte: oxygen ions or protons. SOFC with oxygen-ion-conducting electrolyte are the most developed and have reached the commercialization stage. Usually, the abbreviation SOFC means exactly this type of fuel cell, and it is used without any reservations, but sometimes, when compared with other variants, the oxygen-ion-conducting electrolyte SOFCs are designated O-SOFC [9]. SOFC with proton-conducting electrolyte, which are marked in the literature as H-SOFC or PCFC [9,10], were mentioned already in [4], but their intensive development has been observed only in the last decade.

**Figure 1:**
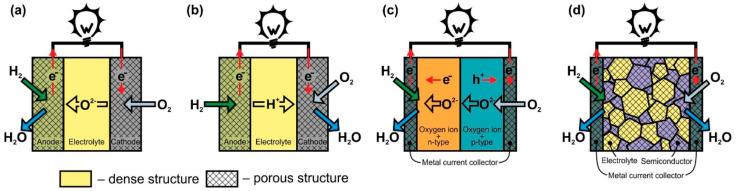
Scheme of operation of (**a**) oxygen-ion conducting electrolyte SOFC (O-SOFC), (**b**) proton-conducting electrolyte SOFC (H-SOFC), (**c**) double-layer fuel cells (DLFC), and (**d**) single-layer fuel cells (SLFC).

In O-SOFC (Figure 1a), oxygen ions move through the electrolyte from the cathode to the anode under the influence of the oxygen chemical potential gradient. To ensure continuous migration of O^2−^ across the electrolyte, the oxygen on the cathode side must enter the electrolyte lattice from the gas phase, leave the electrolyte lattice on the anode side, and react with fuel. The cathodic reaction of converting O_2_ to O^2−^, known as oxygen reduction, involves the absorption of electrons, whereas electrons, H_2_O, and CO_2_ form at the anode when hydrogen or hydrocarbon fuel interacts with O^2−^ supplied by the electrolyte. (Figure 1 shows only hydrogen for simplicity). The electrons released in the fuel oxidation reaction through an external load move to the cathode to participate in the oxygen reduction reaction, thereby generating an electric current.

The open-circuit voltage (OCV) of the fuel cell (when there is no current through the external load) depends on the gradient of the oxygen chemical potential from the cathode and anode sides, and the temperature and pressure in the system [11]. Fuel cell OCV is around 1.1 V at 900 °C with air as oxidant and hydrogen as fuel. When a current is passed, the voltage at the terminals of the fuel cell drops due to its internal resistance, which is the sum of ohmic, polarization, and concentration losses. The voltage on a load can be expressed as:

(1) $$U=E_{OCV}-I\cdot \sum_{i}R_{i}-\eta -\eta_{conc}$$

where *I* is the current, *R_i_* is the ohmic resistance of the SOFC components, *η* is the voltage associated with polarization losses (overpotential), and *η_conc_* is the voltage due to concentration losses. The electrolyte layer makes the main contribution to ohmic losses, since its conductivity is 2–3 orders of magnitude lower than that of electrode materials [4]. Polarization losses (or polarization resistance) are determined by the processes of current formation at the three phase boundary (electrolyte–electrode–gas), which depend on many parameters, such as composition, structure, physicochemical properties of the electrolyte and electrode materials, temperature, and oxygen partial pressure; in addition, they are largely determined by the morphology of the three phase boundary, which, in turn, is set by the prehistory and methods of making electrodes [12]. Concentration losses arise as a result of a change in the reagent concentration in the reaction zone due to the difficulty in delivering reagents (O_2_ and fuel) to the reaction site and the removal of reaction products (H_2_O, CO_2_) through a porous electrode. Concentration losses are small when high porosity and small thickness of the electrodes. The internal resistance of the SOFC should be minimized as much as possible to achieve high specific power.

Most often, oxide materials with a fluorite structure such as Y_2_O_3_ or Sc_2_O_3_ stabilized ZrO_2_ (YSZ or ScSZ, respectively), and Gd_2_O_3_- or Sm_2_O_3_-doped CeO_2_ (GDC or SmDC, respectively) are used as electrolytes for O-SOFC [13,14,15]. In the overwhelming majority of cases, a composite based on Ni is used as an anode material [16,17,18], and the most commonly cathode materials of O-SOFC are La_1-x_Sr_x_MnO_3_ (LSM) [19] and La_1-x_Sr_x_Co_1-y_Fe_y_O_3_ (LSCF) [20]. At the same time, extensive research isbeing conducted to find new electrode materials [6,21,22,23,24,25]. Another area of research that has the prospect of improving SOFC performances is the creation of nanostructures [26,27]. In particular, the introduction of nanosized dense layers with mixed ion-electron conductivity at the cathode–electrolyte interface can significantly reduce the polarization resistance [28,29]. Nanostructuring of electrodes also results in an increase in their catalytic activity and allows the direct use of hydrocarbon fuels [30,31,32,33].

A single SOFC is not suitable for practical use due to its low OCV; therefore, individual cells are connected in a stack to generate a sufficiently high voltage and power. The connection is made using a component called interconnect, which must possess purely electronic conductivity (without oxygen-ion conductivity). The interconnect makes contribution to the internal resistance of the SOFC stack and is an important component together with the anode, cathode, and electrolyte. Consequently, the development of interconnections is also given much attention [6,34,35].

The operation principle of proton-conducting electrolyte SOFC (Figure 1b) is similar to the one of O-SOFC. The difference is that when the fuel is oxidized at the anode, H^+^ enters the electrolyte lattice, and after transferring through the electrolyte, it takes part in the oxygen reduction reaction with the formation of water. It is believed that the formation of water on the cathode side is the advantage of H-SOFC, since, in this case, there is no fuel dilution at the anode. In addition, proton-conducting materials such as SrCeO_3_, BaZrO_3_, and BaCeO_3_ exhibit higher conductivity than that of YSZ or GDC at temperatures of 350–600 °C due to the relatively low activation energy of proton migration in solid oxides [36,37]. Thus, H-SOFC must have a higher power than O-SOFC at low temperatures. However, the properties of the electrolytes and electrodes still have to be improved to completely implement this concept. The main issues associated with the development of proton-conducting electrolytes are to increase chemical stability (prevent interaction with CO_2_ and H_2_O), improve sinterability, and suppress electronic conductivity [38,39]. The greatest hopes for a decrease in polarization losses in H-SOFC are pinned on the development of a cathode material with mixed oxygen-ion-proton-electron triple conductivity [40]. Presently, H-SOFC research is being conducted at the laboratory level with hydrogen as a fuel [38,39].

Electrolyte-free fuel cells can be divided into two classes according to the number of layers of dissimilar materials used to them fabrication: double-layer fuel cells (DLFC) and single-layer fuel cells (SLFC).

The DLFC concept (Figure 1c) was proposed by B. Zhu’s group in [41] and developed in [42]. These are the only publications that we were able to find on this construction. DLFCs are formed from two materials with mixed oxygen-ion and electronic conductivity of n- or p-type. In Ref. [41], the anode and cathode layers were formed from composites to achieve the desired properties of materials. The p-n junction formed at the interface between the two materials prevents the transfer of electrons through the structure of the fuel cell and, in fact, acts as an electrolyte layer with oxygen-ionic conduction in the TL-SOFC.

The SLFC idea was proposed in [43] in 2000. It was based on the assumption that one material can perform the functions of all SOFC components (anode, electrolyte, cathode) due to different types of conductivity at different oxygen partial pressures. The conception was tested on La_0.9_Sr_0.1_InO_3-δ_, which has oxygen ionic conductivity, but at the same time, has p-type conductivity in an oxidizing atmosphere and n-type in a reducing atmosphere. The specific power of Pt/La_0.9_Sr_0.1_InO_3-δ_/Pt cell at 800 °C was 3 mW∙cm^−2^. B. Zhu et al. changed the approach to the formation of SLFC functional layer by making it a porous nanocomposite from materials with oxygen-ionic and semiconducting conduction (Figure 1d) [41,44]. To date, various two- and more-phase composites from materials with different conductivity types have been used for SLFC fabrication. A review of the materials can be found in [8,45]. In particular, ceria–carbonate electrolytes possess H^+^ and O^2−^ conduction [41,46]; therefore, in Figure 1d the formation of H_2_O on the cathode side is shown.

The development of SLFC has been going on for ten years, but the operation principle is still not entirely clear. Two main mechanisms have been proposed to explain the effect of blocking the electron flow through the SLFC functional layer [47,48,49]. The first mechanism consists of the formation of a p-n bulk heterojunction in the center of the composite layer due to the fact that, when exposed to hydrogen and air, electrons and holes concentration zones appear near the fuel and oxidizing current collectors, respectively [47]. The second mechanism is associated with the formation of a Schottky junction between the semiconductor component of the functional layer and the metal current collector on the SLFC anode side [48]. In addition, the role of metal current collectors, which are most often made of Ni and Ag, remains unclear. Do they only serve to transport electrons or also function as electrodes in the SLFC? Nevertheless, it should be recognized that the elimination of the electrolyte layer from the fuel cell design gives the EFFC the following advantages over the conventional three-layer SOFC: (1) ease of manufacture, since only one layer needs to be formed, and (2) the problems of thermomechanical matching of the components are excluded. The developers also declare that polarization losses are reduced because there is no fixed electrode–electrolyte interface. Comparison of the characteristics of SLFC and TL-SOFC made from the same materials indicates that a single-layer structure has similar or even little higher specific power values than a three-layer structure [41,46,48].

A comparison of features of SOFC designs with different electrolytes and without ones is presented in Table 1.

### 2.2. Classification according to the Gas Spaces Separation

**Figure 2:**
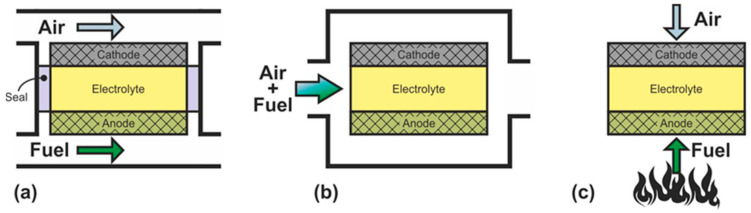
Schematics of (**a**) dual-chamber SOFC, (**b**) single-chamber SOFC, and (**c**) no-chamber SOFC.

SOFC can be divided into three groups according to the criterion of supply of gas reagents: dual-chamber SOFC (DC-SOFC); single-chamber SOFC (SC-SOFC), which are also called “one-chamber”, “mixed-fuels”, or “mixed-reactant”; and no-chamber solid oxide fuel cells, which are most often called direct-flame SOFC (DF-SOFC) or flame fuel cells (FFC) (Figure 2).

In DC-SOFC, the reactants are separated: the oxidant is fed to the cathode, and the fuel is fed to the anode without any mixing (Figure 2a). The operation principle of this configuration was discussed in detail above. It is only recalled that the electromotive force arises by the gradient of the oxygen partial pressure between the separate electrode chambers. Dual-chamber SOFC are considered to the conventional design, and the abbreviation SOFC usually denotes separate-reactant solid oxide fuel cells.

**Figure 3:**
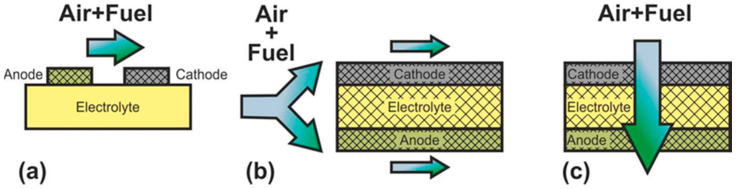
Schematics of (**a**) SC-SOFC with coplanar electrodes and fully porous SOFC in (**b**) flow-by and (**c**) flow-through configuration.

In SC-SOFC, a mixture of fuel and oxidizer is fed into the working chamber (Figure 2b) [50]. In this case, the operation principle is based on the selectivity of the electrodes for the respective reactions. The anode must be electrochemically active for fuel oxidation and inert to oxidant reduction, and the cathode must exhibit selective oxygen reduction and inertness to fuel. The open circuit voltage in SC-SOFC depends on both the electrocatalytic activity and the selectivity of the electrodes. Specific designs of fuel cells can be implemented because there is no need to hermetically isolate the electrodes from each other: SC-SOFC with coplanar electrodes or single-face SC-SOFC and fully porous SOFC (FP-SOFC) or all porous SOFC (Figure 3). In the design with coplanar electrodes (Figure 3a), the electrodes are formed on the same side of the electrolyte, which simplifies the fabrication of SC-SOFC, increases its thermomechanical stability, and allows the formation of several elements at once [51]. In FP-SOFC (Figure 3b,c), the electrolyte layer between the electrodes is porous, which makes the construction cheaper due to low electrolyte sintering temperatures [7]. However, a significant drawback of the specific SC-SOFC designs is the low specific power amounting to 1–40 mW·cm^−2^ at 750 °C [7,52,53]. Only in [54] was a specific power higher than 200 mW·cm^−2^ at 750 °C obtained for FP-SOFC. Moreover, in SOFC with coplanar electrodes, a strong degradation of characteristics is observed [7].

In a mixed-reactant design proposed by M. Horiuchi et al. [55], the fuel cell is placed directly above the burning flame (Figure 2c). The anode is close to the fuel-rich flame, and the cathode has access to ambient air. In this case, the flame provides the fuel cell with heat, carries out the reforming of carbon-hydrogen fuel, and sets the difference in the oxygen partial pressure between the two electrodes by consuming oxygen at the anode. The operation principle of the DF-SOFC is close to the operation principle of the SC-SOFC since gas separation is not required. However, requirements for the selectivity of the catalysts are reduced because the DF-SOFC electrodes are placed in different atmospheres.

The mixed-reactant fuel cells (SC-SOFC and DF-SOFC) have several advantages over DC-SOFC [7,56], especially for small devices. The absence of the need to separate gas spaces results in increased thermomechanical stability and a simpler and compact design both of the fuel cell and the gas manifold. This, in turn, makes the fabrication of a single cell, and its collection in the stack is easier, whereas the formation of necessary effective sealing at high temperatures for separate-reactant SOFC is a challenge [57,58]. Moreover, the rigid connection of the cell to other stack components can result in mechanical stress and even breakage. Another advantage of mixed-reactant SOFC over DC-SOFC is the ability to maintain the operating temperature without the need to supply additional heat from outside. Herewith, DF-SOFC have a number of other advantages: the ability to use almost any hydrocarbon fuel, including gases, liquids, and solids, quick start-up, and the problem of the porous electrodes coking is less serious than that in SC-SOFC.

On the other hand, mixed-reactant SOFC have serious drawbacks that impede their practical use [7,56]. Electrode selectivity plays a key role in the functioning of SC-SOFC. However, fully selective materials have not been found yet. In particular, all SOFC cathode materials catalyze methane oxidation [59]. Due to parasitic reactions occurring at the electrodes, SC-SOFCs have a low electrical efficiency (~1%), as well as a low level of fuel utilization (about 10%) [7]. However, it has recently been shown that the use of a nanocomposite consisting of materials with different functions as a cathode can significantly increase its selectivity and thereby increase the efficiency of the entire SC-SOFC [60]. The electrical efficiency of DF-SOFC is even lower (0.45% [61]), which is associated not so much with the electrode selectivity but with the fact that the fuel is consumed in the combustion reaction. In addition, the significant material stresses arising from thermal load associated with placing the cell near an open flame are a particular problem for DF-SOFC. The use of an air/fuel mixture in SC-SOFC is a risk of ignition and/or explosion. Therefore, in SC-SOFC, hydrogen is not used, and most often, methane is used as a fuel. The separate-reactant SOFC are much safer and have significantly higher electrical efficiency (up to 60% [62]) and a level of fuel utilization (about 80% [62]). Apparently, precisely this huge difference in the efficiency, as well as the immaturity of the technology, are the reason why the mixed-reactant SOFC are not even mentioned in the classifications proposed in [3,4,5,6].

Table 2 summarizes the advantages and disadvantages of DC-SOFC, SC-SOFC, and DF-SOFC.

Currently, DF-SOFC are fabricated based on oxygen-ion-conducting electrolytes [56]. To make SC-SOFC, oxygen-ion conducting electrolytes are also used in most cases [7], but there are single works on the use of proton conducting electrolytes (for example, [63,64]). It is obvious that EFFC operation in the condition of mixed reactants is impossible unless the current collectors possess selectivity to various reactions.

At the end of this section, it is worth mentioning the so-called flame-assisted fuel cells or flame fuel cells (FFC) [65,66,67]. This concept implies two devices integrated with each other: the combustion system and the SOFC itself. The premixed combustion system avoids complete oxidation of the fuel with excess air, which is present in conventional DF-SOFC. As a result, more fuel enters the SOFC anode for electrochemical power generation. Herewith, the air is separately supplied to the fuel cell cathode. Thus, from the point of view of classification, the SOFC operates in a dual-chamber mode. The FFC concept allows the use of a hydrocarbon fuel without any catalysts. However, the efficiency and level of fuel utilization of the FFC are low, although higher than those of the DF-SOFC. The highest electrical efficiency of 6% and fuel utilization coefficient of 23% of FFC have been achieved in [68].

### 2.3. Classification according to Operating Temperature

The first SOFC operated at temperatures of 900–1000 °C [3]. High operating temperatures ensured a low internal resistance of the fuel cell due to the high conductivity of the electrolyte and a high rate of electrode reactions and, accordingly, high specific power as well as the possibility of internal reforming of hydrocarbon fuel. However, high operating temperatures also cause a number of problems related to sealing, the morphological stability of electrodes, the chemical stability of cell components, and the heat resistance of accessories. These problems result in a high cost of cells and a reduction in their lifetime. Therefore, a strategy to reduce the operating temperatures of SOFC was adopted. Lower operating temperatures allow the use of new materials (in particular, steel interconnects [34]), reduce the SOFC cost, reduce degradation, and implement faster start-up.

At the present time, SOFC are usually divided into high-, medium-, and low-temperature categories. However, there is still no consensus on temperature ranges. In works [69,70,71] SOFC are divided only by medium temperature (500–750 °C) and high temperature (above 750 °C). The authors of [72] consider that the definitions of “low-temperature” and “medium-temperature” are a synonyms, and 800 °C is the upper limit of this temperature range. The majority of researchers dividing SOFCs into three temperature classes also define 800 °C as the boundary of medium–high temperatures [6,73,74,75]. Herewith, the boundary between low and medium temperatures varies: in [6,74] and [73,75], 650 and 600 °C are marked, respectively. It should be noted that, usually, the physical reasons for choosing a particular temperature as the range boundary are not explicitly indicated, which, most likely, is the reason for the differences.

The temperature of 800 °C between high- and medium-temperature ranges, accepted by most authors, implies the upper limit of the expediency of using steel interconnects for the stack manufacture [34]. In Ref. [70], it was proposed to make the possibility of implementing internal reforming of methane as a criterion for determining the lower operating temperature of SOFC. The authors of [70] decided that 500 °C is the lowest temperature at which methane internal reforming can occur on a suitable catalyst (catalyst was not specified), although it was recognized that this temperature is controversial. In a recent review [76], it was shown that methane internal reforming at the most common Ni-based anode can occur only at temperatures above 600 °C. Therefore, we propose to set the temperature of 600 °C as the boundary of the medium- and low-temperature region, thereby dividing SOFC into cells that can directly use methane as fuel and cells that require external reforming. Thus, in this work, SOFC operating in temperature ranges above 800 °C, from 600 to 800 °C, and below 600 °C are considered high-, medium-, and low-temperature SOFC, respectively.

The above arguments for lowering the operating temperature generally refer to well-studied separate-reactant O-SOFC. Alternative designs of SLFC, DLFC and H-SOFC have also been developed to reduce operating temperatures. H-SOFC have higher specific powers in the low-temperature region than those of O-SOFC due to more conductive electrolytes. The temperature range of H-SOFC research is 450–750 °C, and the maximum specific power of H-SOFC at 700 °C reaches ~1000 mW∙cm^−2^ [77,78]. The maximum temperature of SLFC testing also did not exceed 750 °C, and the obtained maximum specific powers at 550 °C varied in a wide range, from 200 to 1000 mW∙cm^−2^ [45]. DLFC studies were carried out at temperatures of about 550 °C, and the maximum specific powers were 560 and 280 mW∙cm^−2^ [41,42].

The development of mixed-reactant SOFC was aimed at simplifying the design and did not imply a decrease in operating temperature. The operating temperatures of SC-SOFC vary from 300 to 950 °C [7], and ones of DF-SOFC vary from 400 to 850 °C [79,80,81]. The data on the maximum specific power of SC-SOFC and DF-SOFC presented in the literature have a significant scatter from tens to several hundred mW∙cm^−2^. Their comparison is difficult since the power of mixed-reactant SOFC depends on not only the operating temperature, structure, and materials of the fuel cell, but also on the type of hydrocarbon fuel and the fuel–oxidizer ratio.

### 2.4. Classification according to Support Types

**Figure 4:**
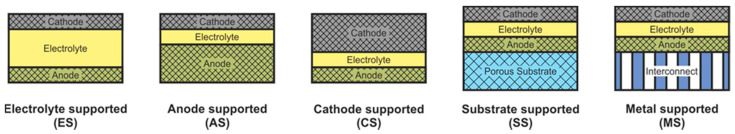
Different types of cell support architectures for SOFC.

SOFC according to supporting component are usually divided into two large groups: self-supporting and external-supporting [5]. In a self-supporting SOFC, one of the components of the fuel cell (electrolyte, anode, or cathode) is the supporting element. In external-supporting SOFC, the supporting element is a porous inert substrate or a metal interconnect. Note that, due to the inherent feature of the design, the supporting substrate of the SC-SOFC can be gas-tight. SOFC schemes with different supporting components are shown in Figure 4.

The advantages and disadvantages of SOFC with different supporting components are presented in Table 3 [5].

The supporting component in the overwhelming majority of the first SOFC was an electrolyte, and its thickness was about 0.2 mm [3,4]. The electrolyte-supported (ES) structure has relatively high strength and therefore has a low probability of mechanical failure, including due to re-oxidation of cermet anode based on Ni. However, the supporting electrolyte layer makes a significant contribution to the internal resistance of the SOFC. Since the conductivity of electrolytes has an exponential dependence on temperature [13], operating temperatures above 800 °C are required to achieve high specific power. Nevertheless, even now, the electrolyte supported design is popular among SOFC manufacturers [82,83,84].

The above-described tendency towards a decrease of the SOFC operating temperature resulted in the need to reduce the electrolyte thickness and transfer the function of the mechanical support to another component. The most widespread at present is the anode-supported (AS) structure of SOFC [85,86,87,88,89], which allows achieving high specific power at temperatures below 800 °C due to the thin electrolyte layer and high conductivity of the Ni-based anode [18]. In addition, this design is cheaper, because the NiO cost is lower than that of electrolyte and cathode materials. However, a significant drawback of the AS design is the possibility of anode re-oxidation, which is accompanied by a volume change of nickel by 41% [16] that can cause mechanical stresses and cell failure. Another problem of electrode supported designs is the limitation of the transfer of gas reagents through the thick, porous layer to the three-phase boundary, which can degrade the characteristics of the cell. In the anode supported structure, this problem is less acute than in the cathode supported (CS) one due to the already mentioned volumetric change of nickel during reduction. Moreover, the conductivity of cathode materials is lower than that of cermet anodes [90], which results in larger internal losses and, accordingly, to a lower specific power of CS SOFC in comparison with AS SOFC. The advantages of the cathode-supported structure include the phase stability of the supporting element (no oxidation–reduction cycles) and low ohmic losses on a thin electrolyte layer. CS SOFCs are not widely used, although this design was used to create the first kW-class generators developed by Simence/Westinghose [91]. It should be noted that the operating temperatures of industrial generators based on CS SOFC were above 900 °C.

The inert substrate-supported (SS) design allows the formation of thin electrolyte and electrode layers. This has to assistant in reducing the operating temperature and reaching of the high specific power of SOFC. Herewith, the supporting substrate can be made of a cheap material that is not usually used in the SOFC. In addition, the supporting substrate can be used as a carrier for a catalyst, allowing the conversion of hydrocarbon fuel into syngas [92]. On the other hand, the introduction of additional material into the SOFC composition increases the complexity of its design and manufacturing technology. Discontinuity of thin functional layers, which is very likely when they are formed on a porous substrate, can result in fuel cell failure. Despite these issues, industrial plants based on SS SOFC have been implemented [93,94] with operating temperatures above 900 °C.

The metal-supported (MS) design is attracting interest because of not only the low operating temperatures and potentially high specific power obtained by thin functional layers but also the high strength and electronic conductivity of the supporting component. However, the development of a technology of MS SOFC fabrication is not an easy task. A high sintering temperature is required for the formation of ceramic materials, while the metal substrate must not be overheated. Other serious problems of this design are the corrosion of the metal substrate under the SOFC operating conditions and the complexity in sealing operations. Advances in the development of interconnect supported SOFC are presented in [95,96]. The target operating temperatures of the MS SOFC are below 800 °C, but there are data on their testing at 850 °C [95].

The overwhelming majority of studies of H-SOFC are carried out on cells having an anode supported construction, which allows obtaining the highest power [39,78,97,98]. Only a few works were performed on cells with a supporting electrolyte [99] and supporting metal [100,101]. All studies of FEFC are currently performed on button cells, which can conditionally be classified as an electrolyte-supported design since the functional layer has a thickness of about 0.5–1 mm. The electrolyte- and anode-supported designs are manly used for DF-SOFC fabrication. A comparison of these two designs carried out in [79] showed that the maximum specific power of AS DF-SOFC (~475 mW∙cm^−2^) is almost four times higher than that of ES DF-SOFC (~121 mW∙cm^−2^). However, there are a few works are devoted to the development of metal supported DF-SOFC [81,102]. In Ref. [81], the peak specific power of 633 mW cm^−2^ was achieved on laboratory samples of MS DF-SOFC, whereas the specific power of the stack prototype tested on a commercial camping stove reached only 156 mW cm^−2^ [102], which is close to the one of ES DF-SOFC. Mostly, electrolytes and anodes are used as supporting components in SC-SOFC too [7,60,103,104,105]. In the single work [106] devoted to cathode-supported SC-SOFC, the peak specific power of only 9 mW∙cm^−2^ was obtained, whereas in AS SC-SOFC, power values of the order of 200–400 mW∙cm^−2^ are achieved [104,105]. A recent numerical simulation [107] has shown that the characteristics of a cathode-supported SC-SOFC should be less than those of an anode-supported SC-SOFC due to the difficulty of oxygen passing through the cathode layer to the reaction zone. The attempt of SC-SOFC forming on supporting dense substrates of MgO and stainless steel, which can be considered a supporting interconnect, was made in [108]. However, the internal resistance of the cells was very high. A numerical estimate of the residence time of the gas mixture in the cell has demonstrated that a structure with a supporting dense substrate for SC-SOFC is impractical [59]. No works describing SC-SOFC with a supporting porous substrate were found.

### 2.5. Classification according to Cell Design

Different geometric shapes of SOFC will be considered at the example of dual-chamber O-SOFC, since this group of fuel cells has the largest number of design options. Discussion of forms of other SOFC types will be carried out based on O-SOFC designs.

**Figure 5:**
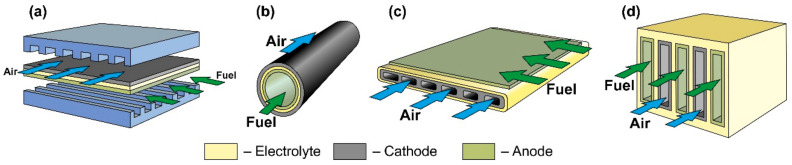
Schematics of (**a**) planar, (**b**) tubular, (**c**) flat-tube, and (**d**) monolithic SOFC.

In accordance with the design, SOFC can be divided into planar, tubular, flat-tubular, and monolithic (Figure 5).

The planar design (Figure 5a) is the most common due to the ease of manufacturing cells and assembling them in a stack, relatively low cost, and high specific volumetric power achieved by close packing of cells and low ohmic losses on interconnects. The disadvantages of the planar design are the difficulty in forming a hermetic seal between the anode and cathode chambers at stack assembly, as well as low resistance to thermal stress. An anode is most often the supporting elements of planar SOFC, but an electrolyte is also a common support. Examples of commercial applications of AS and ES planar SOFC are presented in [70,83]. Planar SOFC with supporting metal are also used in kW class generators [109], whereas the research and development of CS and SS planar cells are carried out at the laboratory level (see, for example, [110,111]).

The tubular SOFC design (Figure 5b) ranks second in popularity [112], although the first stacks were assembled on tubular cells [3,4]. Its advantages include ease of sealing, as well as higher mechanical strength and higher resistance to thermal stress due to the symmetric circular geometry. On the other hand, tubular SOFC have a lower specific volumetric power than planar cells due to less dense packing and larger internal losses associated with long paths of connecting cells in a stack. Moreover, the manufacturing process of the tubular cells is more expensive. Today, as well as for the planar cells, the supporting anode design is the most widespread architecture of tubular SOFC [88], having displaced the cathode-supported design from the leading position. Although, as already mentioned, the first large stacks were assembled on tubular CS SOFC [91]. The tubular design with supporting porous substrate has also been commercially implemented [94], whereas there are few works on ES and IS tubular cells [113,114].

The flat-tube SOFC design (Figure 5c) is essentially a hybrid of planar and tubular ones and it is elaborated to combine the advantages of both SOFC types. In flat-tube SOFC, the sealing is carried out more easily than in a planar design, and the simplicity of assembling cells to a stack is preserved. At the same time, the specific volumetric power of the flat-tube design is higher than that of a tubular one, whereas high resistance to thermal stress is continued. However, the manufacture of flat-tube SOFC should be more complicated and expensive than of planar cells. As the supporting element for a flat-tube design, a cathode (we consider the DELTA design as flat-tube one) [91], an anode [115,116], and a porous substrate [117] are used. A detailed description of materials, fabrication methods, and characteristics of flat-tube SOFC can be found in a recent review [118].

**Figure 6:**
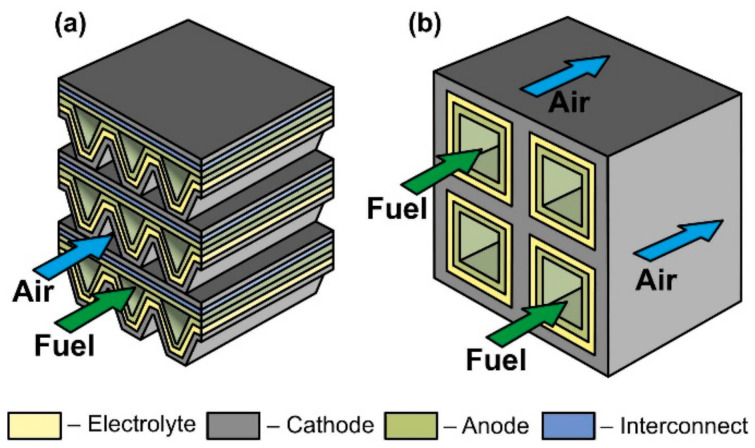
Schematics of (**a**) monolithic stack and (**b**) cathode-supported honeycomb SOFC.

The scheme of a monolithic SOFC design is shown in Figure 5d. It is necessary to clarify that, in works [4,5], where the classification by design was performed jointly for cells and stacks, the term “monolithic design” has a different meaning than in [3]. In Ref. [3], it was believed that the basis of a monolithic fuel cell is a supporting electrolyte with a system of gas channels on the walls of which electrodes are applied (Figure 5d). The electrodes of adjacent channels have opposite signs; therefore, such a monolithic SOFC can be considered a stack of parallel-connected cells. In Refs. [4,5], a monolithic design was meant as a stack assembled from several series-connected corrugated cells that formed gas channels (Figure 6a). In this work, we will adhere to the terminology of [3], since cells and not stacks are classified. In addition, the so-called honeycomb SOFC (Figure 6b) [119,120], which are often distinguished by a special design [75], correspond to the taken definition of a monolithic design. The monolithic design advantages are high thermomechanical strength and a quite high specific volumetric power. However, this design is troublesome to manufacture and has difficulties in sealing and organizing current collectors. Perhaps this is the reason for the small number of works devoted to monolithic SOFC. All investigated monolithic SOFCs had a self-supporting structure, with a supporting electrolyte [119,121], a cathode [120], and an anode [122]. A metal interconnect can be used as a supporting element for a monolithic SOFC, but this will further complicate the fabrication. Applying a supporting porous substrate is apparently impossible without changing the concept of a monolithic SOFC, since the internal contact between adjacent channels will be broken.

Table 4 summarizes the advantages and disadvantages of the four main SOFC designs.

Among planar and tubular SOFC, microplanar and microtubular SOFC are distinguished, the development of which was carried out with an eye to mobile applications. The accentuation of these designs into separate groups is associated not only with the size of the fuel cells but with the features that arise when the size is reduced.

**Figure 7:**
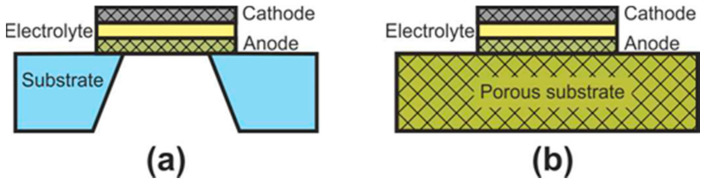
Schematics of (**a**) free-standing FT-SOFC and (**b**) porous substrate supported FT-SOFC.

At the initial development stage of the concept of microplanar SOFC, these cells were usually called simply micro-SOFC [123], whereas now, they are commonly marked to as thin-film SOFC (TF-SOFC) [124,125]. In FT-SOFC, in contrast to large planar cells, the electrolyte layer thickness does not exceed 1 μm, which makes it possible to greatly reduce the operating temperature. The small electrolyte thickness is achieved due to the fact that the electrochemical part of the TF-SOFC is formed on the supporting substrate (Figure 7). There are two configurations of TF-SOFC: free-standing (Figure 7a) and porous substrate supported (Figure 7b).

In free-standing TF-SOFC, an anode–electrolyte–cathode structure is formed over a hole in an inert material substrate (such as a silicon wafer). The main advantage of this structure is the use of very thin electrolytes with a thickness of tens of nanometres [125,126,127] that allows a reduction in the operating temperatures of TF-SOFCs to 300–500 °C. The highest peak power of free-standing TF-SOFCs of 1.3 W∙cm^−2^ at 450 °C [126] was achieved due to the combined effect of using a 60 nm-thick electrolyte and an increased effective area formed due to the three-dimensional architecture of the cell. However, in most works, the peak power is very modest, averaging 200–400 mW∙cm^–2^ [123,125]. In addition, free-standing TF-SOFC have a number of disadvantages: the warping of films during fabrication can result in to cracking; the low mechanical strength of the cathode–electrolyte–anode structure; the small active area of a single cell; manufacturing complexity; and the problem of scaling. Apparently, these drawbacks mean that this type of SOFC is practically not being developed now, although the work [128] proposes a manufacturing method of free-standing metal-supported TF-SOFC. Unfortunately, the cell characteristics are not given.

The fabrication of porous substrate supported TF-SOFCs is much simpler than that of free-standing TF-SOFC and consists of the serial formation of electrode and electrolyte layers on a substrate. The main technical issue at porous substrate supported TF-SOFC fabrication is to avoid the formation of defects in thin functional layers when they are deposited on a rough surface of the substrate. Therefore, material of the substrate is usually either a NiO-based composite, the porosity of which is formed/increased upon nickel reduction [123,125,129,130], or nanostructured anodized aluminum oxide (AAO) [123,125,131]. There are a number of works devoted to the development of metal supported TF-SOFC [132,133]. The values of specific power of porous substrate supported TF-SOFCs vary greatly in the literature, since the cells differ not only in the thickness of the electrolyte but also in the electrode materials. Most often, Pt is used as an electrode due to its low operating temperatures [123,127]. However, the development of nanostructured electrodes allows to abandon noble metals and achieve high SOFC characteristics [134,135,136]. The peak power of ~2.5 W∙cm^−2^ at 650 °C was achieved with a AAO-supported Ni-YSZ | YSZ | GDC | LSCF-YSZ cell with a thickness of about 4 µm [135].

From a classification point of view, a free-standing TF-SOFC is SS SOFC design and a porous substrate supported TF-SOFC can be AS, SS, and MS SOFC depending on the substrate material.

Microtubular SOFC are tubular cells, the outer diameter of which is less than 3 mm. This results in a higher specific volumetric power of the stack and a significant increase in thermal shock resistance [137,138]. Increased thermomechanical characteristics of microtubular SOFC ensure quick start-up and high resistance to thermal cycling. The disadvantages of microtubular SOFC are mainly related to their being assembled in a stack: (1) construction issues at the organization of the current collection and connecting individual cells with each other [139] and (2) sealing of the stack [140]. Nevertheless, in the last decade, microtubular SOFC, due to their advantages, have attracted more attention than standard tubular cells [112,138]. The most common supporting element for microtubular SOFC as well as for tubular cells is an anode [141,142]. Microtubular SOFC with other supporting elements are also researched but there are much fewer works [143,144,145,146]. In addition, nanotube SOFC with an outer diameter of less than 500 nm have been fabricated; however, the obtained specific power was very low (1.3 μW∙cm^−2^ at 550 °C) [147].

Currently, almost all studies of mixed-reactant fuel cells (DF-SOFC and SC-SOFC) have been carried out on button cells. There are only a few works on the use of microtubes to investigate SOFC in a single-chamber regime [148]. H-SOFCs are also studied mainly in the form of button cells [38,39]; however, there are several works to obtain sufficiently large anode supported planar cells [149,150]. In addition, AS H-SOFC were fabricated in tubular [151,152], thin film [153], and microtubular [78,154,155] designs.

## 3. Systematization of SOFC

**Figure 8:**
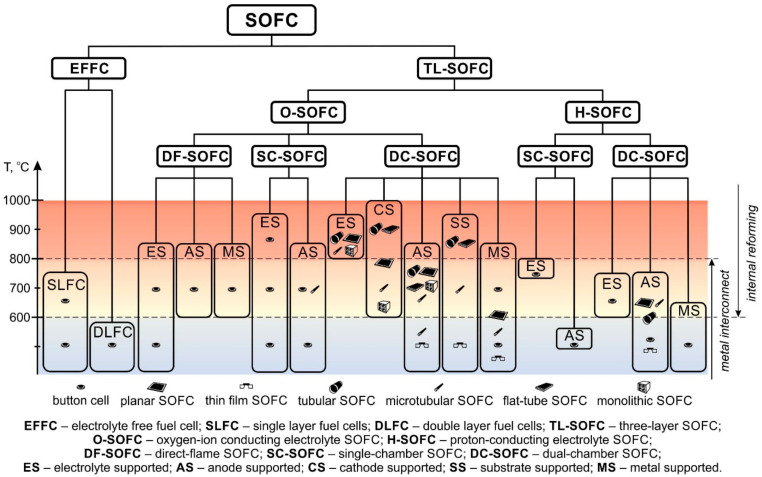
Systematization of SOFC types.

Figure 8 shows a summary scheme of all types of SOFC realized to date. It should be emphasized that the previously considered SOFC divisions are equal, and the presented scheme does not have a strict hierarchical structure. The established dominance of the attributes by which the division was made is rather arbitrary, but we hope that this scheme clearly reflects the current state of developments in the SOFC field. The difference between the “button cell” and “planar SOFC” icons is the size of the cells. “Button cell” denotes small samples used in laboratory research, and “planar SOFC” denotes a large planar cell (linear dimension of several cm) suitable for making stacks. It can be seen that EFFC, mixed-reactant O-SOFC, and H-SOFC are still at the development stage, whereas dual-chamber O-SOFC are already used to manufacture industrial generators. Unfortunately, it is impossible to reflect in the scheme the number of works devoted to a particular type of SOFC design and, consequently, its demand. Single works concern DLFC [41,42], single-chamber H-SOFC [63,64], ES and MS H-SOFC [99,100] and CS O-SOFC [91,110,120,144], whereas the fabrication and testing of ES and AS O-SOFC is described in thousands of works. The temperature range for each type of SOFC in the scheme covers all data presented in the literature. On the one hand, this shows the temperature borders within which a certain SOFC type can function. On the other hand, this does not give a representation of the optimal operating temperature of this SOFC type. For example, the upper border of the SLFC operating range was set at 750 °C in accordance with [46], although studies of the characteristics of SLFC have mainly been carried out at 550 °C. Nevertheless, Figure 8 shows that the most well-developed O-SOFC used industrial generators operate at temperatures of 800 °C and above. Only AS O-SOFC stacks operate at lower temperatures [88,115]. Alternative designs such as EFFC and H-SOFC are promising for reducing operating temperatures; however, research and development are needed to reach a more mature state of these technologies.

## 4. Separate Designs and Concepts of SOFC

Several separated SOFC designs and concepts which do not mention earlier are presented in the literature. At the end of the review, these types of SOFC will be briefly considered, and their place in the proposed classification will be defined.

**Figure 9:**
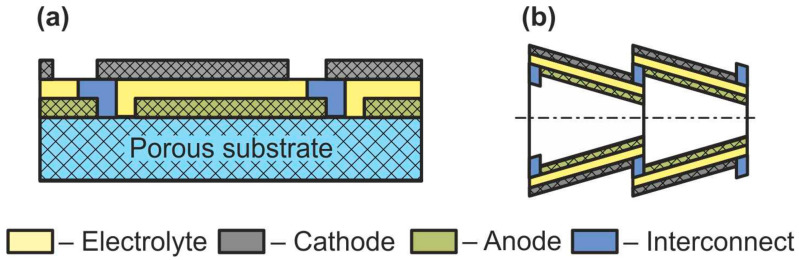
Schematics of (**a**) segmented-in-series SOFC and (**b**) cone-shaped SOFC.

Sometimes, together with flat-tube and honeycomb SOFC, such designs as segmented-in-series or integrated planar SOFC and cone-shaped SOFC are discussed (Figure 9) [75]. Short current collectors inherent in these designs allow reducing the weight and cost of a fuel cell as well as improving its performance by low ohmic losses associated with the connection of the cells with each other. However, it must be emphasized that this is not some new type of separate cell design but a technique for connecting cells into a stack. The cone-shaped cells are a kind of tubular SOFC, whereas the segmented-in-series design was fabricated in both flat [93] and tubular [94] geometries.

The concept of symmetric SOFC (SSOFC) is to replace different electrode materials (anodic and cathodic) of conventional SOFC on one material [156]. This simplifies and reduces the cost of fabricating fuel cells, since both electrodes can be fired in one thermal cycle. In addition, the use of the same material for the anode and cathode diminishes the problems of thermomechanical compatibility of SOFC components by the formation of the same electrode–electrolyte boundaries. Another advantage of the symmetric SOFC concept is the ability to solve the issues associated with sulfur poisoning and carbon deposition by changing the direction of gas flows to oxidize these substances. Any design of a dual-chamber SOFC is suitable for the implementation of SSOFC concept, since the term “symmetric” means the same electrode materials and not the configuration of the cell itself. However, mixed-reactant SOFC with identical electrodes will not function, since it is impossible for one material to have selectivity to different reactions. The development of an electrode material that must simultaneously satisfy all the requirements for cathode and anode of SOFC is an obstacle to the realization of symmetric SOFC [156,157,158].

Another noteworthy concept is reversible SOFC (RSOFC or RSOC), which implies that a solid-state electrochemical device can operate both in the fuel cell mode and in the electrolysis mode [159,160]. In the first mode, RSOFC operates on the SOFC principle converting fuel into electricity. In the second mode, the RSOFC operates as a solid oxide electrolysis cell (SOEC), consuming energy and generating hydrogen (fuel) from water. Thus, RSOFCs can “preserve” excess electricity in the form of chemical energy of the produced substances (mainly hydrogen) and, if necessary (during peak electricity demand), convert the fuel back into electricity. As in the case of SSOFC, RSOFC can be implemented in any separate-reactant SOFC design. Today, pilot plants of RSOFC are already being tested [160]. However, a number of problems still need to be solved for the commercialization of RSOFC: an exact understanding of cell behavior and its degradation when switching modes, the selection of materials and operating parameters suitable for reversible operation, a connection of RSOFC to existing networks, and reducing the cost.

## 5. Conclusions

A brief description of all SOFC configurations developed today is presented in the review. To cover all SOFC concepts, the standard SOFC classification is supplemented by division according to such criteria as presence/absence of electrolyte and gas spaces separation. Herewith, the types of SOFC that are usually not mentioned in the classifications (electrolyte-free fuel cell and mixed-reactant SOFC) have been considered along with other types of SOFC from standpoint of standard criteria: operating temperature, support types, and geometry. This has made it possible to compare the various designs. It is shown that the most developed group of SOFC are separate-reactant fuel cells with oxygen-ion-conducting electrolytes. Among them, the most popular design is an anode-supported one, which permits one to achieve high specific powers at temperatures below 800 °C. However, electrolyte-free SOFC and proton-conducting electrolyte SOFC, the intensive development of which began recently, have a greater potential for reducing operating temperatures than standard dual-chamber O-SOFC. All SOFC types have some drawbacks; therefore, further research and new ideas are necessary for the practical mass implementation of this technology.

## Figures and Tables

**Table 1:** Features of O-SOFC, H-SOFC, and FEFC.

SOFC Type	Advantages	Disadvantages
O-SOFC	Well-studied There are industrial devices Potential for internal reforming	Complexity of fabrication Limited selection of materials Low conductivity electrolyte High operating temperatures result in higher thermomechanical stresses and more significant degradation
H-SOFC	Higher conductive electrolyte Low operating temperatures suggest less thermomechanical stress and less degradation No fuel dilution with reaction products (H_2_O)	More research on electrolyte and electrode materials are required Complexity of fabrication Internal reforming is questionable
DLFC	Simplicity of fabrication The problem of thermomechanical matching of cell materials is alleviated Wide selection of materials	Poorly studied No internal reforming
SLFC	Simplicity of fabrication No problem with thermomechanical matching of cell materials Wide selection of materials	Poorly studied Internal reforming is questionable

**Table 2:** Features of DC-SOFC, SC-SOFC, and DF-SOFC.

SOFC Type	Advantages	Disadvantages
DC-SOFC	Well-studied There are industrial devices High efficiency High level of fuel utilization Fire and explosion safety	Complexity of fabrication Matching of thermal expansion of cell materials are required Slow start up
SC-SOFC	Simplicity of fabrication Simplified use of hydrocarbons as fuel High resistance to thermomechanical stress	More selective electrodes are required Low efficiency Low level of fuel utilization Flammable and explosive Coking of electrodes
DF-SOFC	Simplicity of fabrication Simplified use of hydrocarbons as fuel Potential for quick start up	More selective electrodes are required Low efficiency Low level of fuel utilization High thermomechanical stress Coking of electrodes

**Table 3:** Features of SOFC with the different supporting components.

**SOFC Type**	**Advantages**	**Disadvantages**
Self-supporting		
ES SOFC	Relatively strong structural support from dense electrolyte Less susceptible to failure due to anode reoxidation (Ni/YSZ anode) and cathode reduction (LSM cathode)	Higher resistance due to low electrolyte conductivity Higher operating temperatures required to minimize electrolyte ohmic losses
AS SOFC	Highly conductive anode Lower operating temperature via use of thin electrolytes	Potential anode reoxidation Mass transport limitation due to thick anodes
CS SOFC	No oxidation issues but potential cathode reduction Lower operating temperature via use of thin electrolyte	Lower conductivity Mass transport limitation due to thick cathodes
External-supporting		
SS SOFC	Thin cell components for lower operating temperature Potential for use of non-cell material for support to improve properties	Increased complexity due to addition of new materials Possibility of formation of discontinuous layers on a porous substrate
MS SOFC	Thin cell components for lower operating temperature Stronger structures from metallic interconnects	Interconnect oxidation Flowfield design limitation due to cell support requirement

**Table 4:** Features of the different SOFC designs.

SOFC Type	Advantages	Disadvantages
Planar	High power density Simplicity of stack assembly	Low resistivity of thermomechanical stress Difficulties with sealing
Tubular	The resistivity of thermomechanical stress Sealing is simpler than that of planar SOFC	Low power density High internal resistance
Flat-tube	The resistivity of thermomechanical stress Simplicity of stack assembly	Complexity of fabrication of single cell High internal resistance
Monolithic	Sufficiently high power density High thermomechanical strength High durability	Complexity of fabrication Difficulties with the formation of the current contacts Difficulties with sealing

## Data Availability

Not applicable.
